# Analysis of Hybrid NAR-RBFs Networks for complex non-linear Covid-19 model with fractional operators

**DOI:** 10.1186/s12879-024-09329-6

**Published:** 2024-09-27

**Authors:** Aqeel Ahmad, Muhammad Farman, Muhammad Sultan, Hijaz Ahmad, Sameh Askar

**Affiliations:** 1 Department of Mathematics, Ghazi University, DG Khan, 32200 Pakistan; 2 Department of Mathematics, Khawaja Fareed University of Engineering and Information Technology, Rahim Yar Khan, Pakistan; 3 Department of Computer Science and Mathematics, Lebanese American University, Beirut, Lebanon; 4 Department of Mathematics, Sait University, Calgary, Canada; 5 Operational Research Center in Healthcare, Near East University, Nicosia/TRNC, 99138 Mersin 10, Turkey; 6 Department of Mathematics, College of Science, Korea University, 145 Anam-ro, Seongbuk-gu, Seoul 02841, South Korea; 7 Department of Technical Sciences, Western Caspian University, Baku 1001, Azerbaijan; 8 Department of Statistics and Operations Research, King Saud University, Riyadh, Saudi Arabia

**Keywords:** NAR-RBFs Networks, Boundedness, Stability, Uniqueness, Mittag-Leffler kernel

## Abstract

The Hybrid NAR-RBFs Networks for COVID-19 fractional order model is examined in this scientific study. Hybrid NAR-RBFs Networks for COVID-19, that is more infectious which is appearing in numerous areas as people strive to stop the COVID-19 pandemic. It is crucial to figure out how to create strategies that would stop the spread of COVID-19 with a different age groups. We used the epidemic scenario in the Hybrid NAR-RBFs Networks as a case study in order to replicate the propagation of the modified COVID-19. In this research work, existence and stability are verified for COVID-19 as well as proved unique solutions by applying some results of fixed point theory. The developed approach to investigate the impact of Hybrid NAR-RBFs Networks due to COVID-19 at different age groups is relatively advanced. Also obtain solutions for a proposed model by utilizing Atanga Toufik technique and fractal fractional which are the advanced techniques for such type of infectious problems for continuous monitoring of spread of COVID-19 in different age groups. Comparisons has been made to check the efficiency of techniques as well as for finding the reliable solutions to understand the dynamical behavior of Hybrid NAR-RBFs Networks for non-linear COVID-19. Finally, the parameters are evaluated to see the impact of illness and present numerical simulations using Matlab to see actual behavior of this infectious disease for Hybrid NAR-RBFs Networks of COVID-19 for different age groups.

## Introduction

COVID-19 (SARS-CoV-2) is one of the biological problem which is a lethal pandemic that has spread over the world during the last quarter of 2019. In recommendation to the early 2003 wave, which was provoke by a corona virus. This disease can transmit from one biological organisms to another and has immediate effect. It is transmitted from one to another human being through coughing, sneezing, talking, breathing etc. which are presented in the air. Other human beings are effected by the close touch with infected persons or by touching things or gadgets. Later by touching their eyes, noses and mouths without washing their hands [1, 2]. Generally, the disease indication increased from 5-7 days after infection and it reaches its extreme from 2-12 days. To avoid its drenched the effected person must be separated for 14 days. For complete understanding of this unseen transmission and incubation the length among preliminary meet until the virus, and the first sign was located [3]. Researcher in Europe predict that Covid-19 will spread in France probably in mid-January. But this prediction proved not true because the ratio of spreading this virus was very low in France and its surrounding regions [4]. People more than 70 years who were infected with COVID-19 included having other disease as heart sickness, lung disease, cancer and diabetes etc. caused their death [5]. But the number of infected individuals were increasing day by day [6, 7]. COVID-19 is socialize in a way that a number of people were infected without knowing its signs.

The way of spreading of this virus was disorderly along with rapid expansion. Three main reason for rapid spreading were density of population, less average duration of infection and simple way of spreading virus. In this regard investigation is taken out in [8]. The core intention to search out who and how a human being infected by someone in [9]. The debatable thing to find out sign and symptoms from which an individual is infected where is a difference in regarding the duration of spreading this virus between American observer and Chines Observer [10]. The first case of Covid-19 was found on 10th March 2020 Cango after 6 weeks there were more than 400 peoples were found affected by this virus in United States and after 3 months it increase more than 5000 affected persons [11]. All these situation depict how rapidly this disease is spreading especially in some countries. The study show how this disease was spread in Cango. This situation show the similarities with upward and downward slopes of the plague.

The index case of this mater might have an influence on the contact instances, therefore, debates should be open on this topic. Several mathematical models were chalked out by the researcher to indicate how the infections spread specially Covid-19 [12–18]. A number of instances were analyzed to encounter this pandemic [19]. Mathematical model show that the sea food market paly the basic role in expansion of Covid-19 [20]. Mathematical versions provide parameters to check Covid-19 and also point out the parameters which are helpful to control the pandemic [21]. The suggested model of Atangana-Baleanu-Caputo (ABC) derivate is helpful for both instances like that healthy and infected [22]. A fixed point theory also support Atangana-Baleanu-Caputo (ABC) derivative with fractional order [23]. Fractional ABC operator is primary base on mathematical version [24–26]. A version having four elements (vulnerable, uncovered, infected and recovered) are discuss in [27]. Also the COVID-19 with vaccination effects and by employing different tools are given [28, 29]. The stochastic modeling on COVID-19 and its related type infectious diseases are investigated in [30–32] by different mathematical tools.

In recent years many definitions of fractional derivatives have been prepared and worked out to produce a mathematical model for real word system. Main purpose of current work is to develop and analysis Atangana-Baleanu Caputo for fractional derivatives model of COVID-19 pandemic as well as fractal fractional technique to see the spread of COVID-19 in different age groups as well as for continuous monitoring. The unique and bounded solution of the fractional order system is established by using fixed point theory and iterative methods. The effects of different parameter are shown graphically using MATLAB Coding. For the numerical result of the COVID-19 model concerned with advanced ABC derivative is compared with classical result for COVID-19 model using different fractional parameters as well as with fractal fractional approach for reliable findings.

## Preliminaries

This section consist of basic concept of sammudu transform and fractional DEs described in [27, 33, 34].

### Definition 1

The fractional-order derivative of ABC in Liouville-Caputo sense is mentioned as

$$\begin{aligned} ^{ABC}_{\gamma _{1}}D^{\gamma _{1}}_{t}\{f(t)\}=\frac{AB(\gamma _{1})}{m-\gamma _{1}}\int ^{t}_{\gamma _{1}}\frac{d^{m}}{dw^{m}}f(w)E_{\gamma _{1}}\left[ -\gamma _{1}\frac{(t-w)^{\gamma _{1}}}{m-\gamma _{1}}\right] dw, m-1<\gamma _{1}<m, \end{aligned}$$

where $E_{\gamma _{1}}$ is the Mittag-Leffler function and $AB(\gamma _{1})$ is a normalization function and $AB(0)=AB(1)=1.$

### Definition 2

A sumudu transform for the function $\psi (z)$

$$\begin{aligned} S = {\psi (z): \exists ~ \hbar ,~ \chi _1, \chi _2 >0, ~ ~ \psi (z) < \hbar ~ exp \left( \frac{|\chi |}{\chi _1}\right) , if ~ ~ z \in (-1)^j \times [0,\infty )} \end{aligned}$$

can be expressed as

$$\begin{aligned} F(v)=ST[\psi (z)]=\int ^{\infty }_0 exp(-\chi )\psi (v\chi )d\chi , v\in (-\chi _1, \chi _2. \end{aligned}$$

### Definition 3

Antagana-Baleanu is defined as

$$\begin{aligned} {}_{\alpha }^{ABC} D_{t}^{\alpha } (\psi (t)) = \frac{AB(\alpha )}{n-\alpha }\int ^{\chi }_\alpha \frac{d^n}{dw^n} f(w)E_\alpha {-\alpha \frac{(\chi -w)^\alpha }{n-\alpha }}dw, ~ n-1<\alpha <n, \end{aligned}$$

For the above equation, using laplace transformation, we have:

$$\begin{aligned} L\left[{}_{\alpha }^{ABC} D_{t}^{\alpha } (\psi (t))\right](S) = \frac{AB(\alpha )}{1-\alpha }\frac{S^\alpha L[\psi (\tau )](S)-S^{\alpha -1}\psi (0)}{S^\alpha + \frac{\alpha }{1-\alpha }}. \end{aligned}$$

applying ST for the above equation, we get

$$\begin{aligned} ST\left[{}_{0}^{ABC} D_{t}^{\alpha } (\psi (t))\right](S) = \frac{B(\alpha )}{1-\alpha +\alpha S^\alpha }\times [ST \psi (t)-\psi (0)]. \end{aligned}$$

## Materials and method

In this epidemic model $S_1-S_2-I-T-R$ given in [35], we divide the all population into five time-dependent classes, such as $S_1(t)$ represent those persons who are uninfected; $S_2 (t)$ be the persons have some kinds of sickness/older age; *I*(*t*) represents the infected persons by Covid-19; *T*(*t*) represents the persons which are under the treatment in a hospital or the state of quarantined; *R*(*t*) represents the persons which are recovered with treatment. Thus, the following five differential equations represent the mathematical model.

1 $$\begin{aligned} \frac{dS_{1}}{dt}=B-\beta \times I(t)\times S_{1}(t) -\delta \times \beta \times T(t)-\alpha \times S_{1}(t) \end{aligned}$$

2 $$\begin{aligned} \frac{dS_{2}}{dt}=B-\beta \times I(t)\times S_{2}(t)-\delta \times \beta \times T(t)-\alpha \times S_{2}(t) \end{aligned}$$

3 $$\begin{aligned} \frac{dI}{dt}=- \mu \times I(t)+\beta \times I(t)\times [S_{1}(t)+S_{2}(t)]-\alpha \times I(t)+\beta \times \delta \times T(t)+\sigma \times I(t) \end{aligned}$$

4 $$\begin{aligned} \frac{dT}{dt}=\mu \times I(t)-\rho \times T(t)-\alpha \times T(t)+\psi \times T(t)+\varepsilon \times T(t)) \end{aligned}$$

5 $$\begin{aligned} \frac{dR}{dt}=- \alpha \times R(t)+\rho \times T(t) \end{aligned}$$

with initial coditions. $S_{1}(0)=S_{1(0)}$ , $S_2(0)=S_{2(0)}$ , $I(0)=I_{0}$, $T(0)=T_0$ , $R(0)=R_0$ In mathematical models, all parameter values are labeled as $\beta$ represents Contact rate, B is a rate of Natural birth, $\delta$ represents decreased sepsis from the medicament, $\sigma$ represents high temperature and rate of dry cough, $\mu$ is a rate Recovery, $\alpha$ is the Death rate, $\rho$ represents infection rate from the medication, $\psi$ represents the rate of Healthy food, $\varepsilon$ is the Sleeping rate.

Mathematical model for COVID-19 in Antagana-Baleanu Caputo define is as follows.

6 $$\begin{aligned} ^{ABC}_{0}D^{\gamma }_{t} S_{1}=B-\beta I\times S_{1}-\delta \times \beta \times T-\alpha \times S{1} \end{aligned}$$

7 $$\begin{aligned} ^{ABC}_{0}D^{\gamma }_{t}S{2}=B-\beta \times I\times S_{2} -\delta \times \beta \times T-\alpha S_{2} \end{aligned}$$

8 $$\begin{aligned} ^{ABC}_{0}D^{\gamma }_{t}I=- \mu \times I+\beta \times I\times [S_{1}+S_{2}]-\alpha \times I+\beta \delta \times T+\sigma \times I \end{aligned}$$

9 $$\begin{aligned} ^{ABC}_{0}D^{\gamma }_{t}T=\mu \times I-\rho \times T-\alpha \times T+\psi \times T+\varepsilon \times T \end{aligned}$$

10 $$\begin{aligned} ^{ABC}_{0}D^{\gamma }_{t}R=- \alpha R+\rho T \end{aligned}$$

Here $^{ABC}_{0}D^{\gamma }_{t}$ shows the fractional derivative of Antagana-Baleanu Caputo sense, and $0< \gamma \le 1$. Initial conditions of this system’s is: $S_{1}(0)=S_{1(0)}$ , $S_2(0)=S_{2(0)}$ , $I(0)=I_{0}$ , $T(0)=T_0$ , $R(0)=R_0$

## Stability analysis by iterative method

Features of the inner product and Hilbert space, and utilizing fixed point theory. In special solutions, we show the uniqueness and present a detailed analysis of stability in this approach. Consider (*L*, |.|) is the Banach space and H be the mapping of L. Let us suppose $z_{n+1}= g(H, z_{n})$ be a specific Repetition and repetitive way. These situation is satisfy for $z_{n+1}=H z_{n}$.

$*$ which is at least one element exists in H’s fixed point set.

$*$ $z_{n}$ is converges to $P\epsilon F(H)$. $*$ $\lim _{n\rightarrow \infty } x_{n}(t)=P$.

### Theorem 1

Let (*L*, |.|) is a Banach space and H a self-maping of L satisfying

$$\begin{aligned} \Vert H_{l}-H_{r}|\le \theta \Vert H-H_{l}|+\theta \Vert l-r| \end{aligned}$$

for all $l,r\epsilon L$,where $0\le \theta \le 1$. Assume that H is Picard in H-stable.

Take a look at the recursive formula which is taken from approximate solution of the system by applying the sumudu transform operator. The operator is applied to both sides of Eqs. (6)-(10) as follows.

11 $$\begin{aligned} S_{1(n+1)}=S_{1(n)} (0)+ST^{-1} \frac{1-\gamma }{A(\gamma )\gamma \Gamma (\gamma +1)E_{\gamma }\left(-\frac{1}{1-\gamma }\omega ^{\gamma }\right)}\times ST[B-\beta I\times S_{1}(t) -\delta \beta T-\alpha S_{1}] \end{aligned}$$

12 $$\begin{aligned} S_{2(n+1)}=S_{2(n)} (0)+ST^{-1} \frac{1-\gamma }{A(\gamma )\gamma \Gamma (\gamma +1)E_{\gamma }\left(-\frac{1}{1-\gamma }\omega ^{\gamma }\right)}\times ST[B-\beta I\times S_{2} -\delta \beta T-\alpha S_{2}] \end{aligned}$$

13 $$\begin{aligned} I_{n+1} =I_{n} (0)+ST^{-1} \frac{1-\gamma }{A(\gamma )\gamma \Gamma (\gamma +1)E_{\gamma }\left(-\frac{1}{1-\gamma }\omega ^{\gamma }\right)}\times ST[- \mu I+\beta I\times [S_{1}+S_{2}]-\alpha I+\beta \delta T+\sigma I] \end{aligned}$$

14 $$\begin{aligned} T_{n+1} =T_{n} (0)+ST^{-1} \frac{1-\gamma }{A(\gamma )\gamma \Gamma (\gamma +1)E_{\gamma }\left(-\frac{1}{1-\gamma }\omega ^{\gamma }\right)}\times ST[\mu I-\rho T-\alpha T+\psi T+\varepsilon T] \end{aligned}$$

15 $$\begin{aligned} R_{n+1} =R_{n} (0)+ST^{-1} \frac{1-\gamma }{A(\gamma )\gamma \Gamma (\gamma +1)E_{\gamma }\left(-\frac{1}{1-\gamma }\omega ^{\gamma }\right)}\times ST[- \alpha R+\rho T] \end{aligned}$$

these equations linked with the fractional Lagrange multiplier.

### Proof

Defining H as a self-map is as follows:

16 $$\begin{aligned} H[S_{1(n+1)}]=S_{1(n+1)} =S_{1(n)} (0)+ST^{-1}\{\frac{1-\gamma }{A(\gamma )\gamma \Gamma (\gamma +1)E_{\gamma }\left(-\frac{1}{1-\gamma }\omega ^{\gamma }\right)} \times ST[B-\beta I\times S_{1} -\delta \beta T-\alpha S_{1}]\} \end{aligned}$$

17 $$\begin{aligned} H[S_{2(n+1)} ] =S_{2(n+1)}=S_{2(n)} (0)+ST^{-1}\left\{ \frac{1-\gamma }{A(\gamma )\gamma \Gamma (\gamma +1)E_{\gamma }\left(-\frac{1}{1-\gamma }\omega ^{\gamma }\right)} \times ST\left[B-\beta I S_{2} -\delta \beta T-\alpha S_{2}\right]\right\} \end{aligned}$$

18 $$\begin{aligned} H[I_{n+1} ]=I_{n+1}=I_{n} (0)+ST^{-1} \left\{\frac{1-\gamma }{A(\gamma )\gamma \Gamma (\gamma +1)E_{\gamma }\left(-\frac{1}{1-\gamma }\omega ^{\gamma }\right)} \times ST\left[- \mu I +\beta I [S_{1}+S_{2}]-\alpha I+\beta \delta T+\sigma I\right]\right\} \end{aligned}$$

19 $$\begin{aligned} H[T_{n+1}]=T_{n+1} =T_{n} (0)+ST^{-1}\left\{ \frac{1-\gamma }{A(\gamma )\gamma \Gamma (\gamma +1)E_{\gamma }\left(-\frac{1}{1-\gamma }\omega ^{\gamma }\right)} \times ST\left[\mu I-\rho T-\alpha T+\psi T+\varepsilon T\right]\right\} \end{aligned}$$

20 $$\begin{aligned} H[R_{n+1} ]=R_{n+1} =R_{n} (0)+ST^{-1}\left\{ \frac{1-\gamma }{A(\gamma )\gamma \Gamma (\gamma +1)E_{\gamma }\left(-\frac{1}{1-\gamma }\omega ^{\gamma }\right)} \times ST\left[- \alpha R+\rho T\right]\right\} \end{aligned}$$

Using norm properties and accounting for triangular inequality, so

$$\begin{aligned} \Vert H[S_{1(n)}]-H[S_{1(m)}]||\le \Vert S_{1(n)}-S_{1(m)}||+ST^{-1}\left\{{\frac{1-\gamma }{A(\gamma )\gamma \Gamma (\gamma +1)E_{\gamma }\left(-\frac{1}{1-\gamma }\omega ^{\gamma }\right)}}\times ST\left[B-\beta (I_n \times S_{1(n)}-I_m \times S_{1(m)})-\delta \beta (T_n-T_m)-\alpha (S_{1(n)}-S_{1(m)})\right]\right\} \end{aligned}$$

$$\begin{aligned} \Vert H[S_{2(n)}]-H[S_{2(m)}]||\le \Vert S_{2(n)}-S_{2(m)}||+ST^{-1}\left\{{\frac{1-\gamma }{A(\gamma )\gamma \Gamma (\gamma +1)E_{\gamma }\left(-\frac{1}{1-\gamma }\omega ^{\gamma }\right)}}\times ST\left[B-\beta (I_n\times S_{2(n)}-I_m \times S_{2(m)})-\delta \beta (T_n-T_m)-\alpha (S_{2(n)}-S_{2(m)})\right]\right\} \end{aligned}$$

$$\begin{aligned} \Vert H[I_{n}]-H[I_{m}]||\le \Vert I_{n}-I_{m}||+ST^{-1}\left\{{\frac{1-\gamma }{A(\gamma )\gamma \Gamma (\gamma +1)E_{\gamma }\left(-\frac{1}{1-\gamma }\omega ^{\gamma }\right)}}\times ST\left[- \mu ( I_n-I_m )+\beta ( I_n (S_{1(n)}+S_{2(n)})- I_m (S_{1(m)}+S_{2(m)})) -\alpha (I_n-I_m)+\beta \delta (T_n-T_m)+(\sigma _n-\sigma _m)\right]\right\} \end{aligned}$$

$$\begin{aligned} \Vert H[T_{n}]-H[T_{m}]||\le \Vert T_{n}-T_{m}||+ST^{-1}\left\{{\frac{1-\gamma }{A(\gamma )\gamma \Gamma (\gamma +1)E_{\gamma }\left(-\frac{1}{1-\gamma }\omega ^{\gamma }\right)}}\times ST\left[\mu ( I_n-I_m)-\rho ( T_n-T_m)-\alpha ( T_n-T_m)+\psi ( T_n- T_m)+ \varepsilon (T_n-T_m)\right]\right\} \end{aligned}$$

$$\begin{aligned} \Vert H[R_{n}]-H[R_{m}]||\le \Vert R_{n}-R_{m}||+ST^{-1}\left\{{\frac{1-\gamma }{A(\gamma )\gamma \Gamma (\gamma +1)E_{\gamma }\left(-\frac{1}{1-\gamma }\omega ^{\gamma }\right)}}\times ST\left[- \alpha ( R_n-R_m)+\rho ( T_n-T_m)\right]\right\} \end{aligned}$$

When H satisfies the conditions outlined in Theorem 1,

$$\begin{aligned} \theta =(0,0,0,0,0),\theta =\left\{ \begin{array}{ccccc} ||S_{1(n)}-S_{1(m)}|| \times ||-S_{1(n)}+S_{1(m)}||\\ +B-\beta ||I_n. S_{1(n)}-I_m.S_{1(m)}||-\delta \beta ||T_n-T_m||\\ -\alpha ||S_{1(n)}-S_{1(m)}||\\ \times ||S_{2(n)}-S_{2(m)}|| \times ||-S_{2(n)}+S_{2(m)}||\\ +B-\beta ||I_n S_{2(n)}-I_m S_{2(m)}||-\delta \beta ||T_n-T_m||\\ -\alpha ||S_{2(n)}-S_{2(m)}||\\ \times ||I_{n}-I_{m}||\times ||-I_{n}+I_{m}||\\ -\mu || I_n-I_m ||+\beta || I_n (S_{1(n)}+S_{2(n)})- I_m (S_{1(m)}+S_{2(m)})||\\ -\alpha ||I_n-I_m||+\beta \delta ||T_n-T_m||+||\sigma _n-\sigma _m||\\ \times ||T_{n}-T_{m}||\times ||-T_{n}+T_{m}||\\ + \mu || I_n-I_m||-\rho || T_n-T_m||-\alpha || T_n-T_m||\\ +\psi ||T_n-T_m||+\varepsilon ||T_n-T_m||\\ \times ||R_{n}-R_{m}||\times ||-R_{n}+R_{m}||\\ - \alpha || R_n-R_m||+\rho || T_n-T_m|| \end{array}\right\} \end{aligned}$$

And We also mention that H is Picard’s H-stable. The proof is now complete.

## Uniqueness of the special solution

### Theorem 2

The special solution of Eqs. (3)-(10), the iteration approach provide a unique singular solution.

The Hilbert space is taken into consideration. $H=L^{2}((x,y)\times (0,r))$ that can be define as the set of these function:

$$\begin{aligned} f:(x,y)\times [0,T]\rightarrow R,\int \int \int gfdgdf<\infty \end{aligned}$$

This operator as follow

$$\begin{aligned} \theta =(0,0,0,0,0),\theta =\left\{ \begin{array}{ccccc} B-\beta I.S_{1}-\delta \beta T-\alpha S_{1}\\ B-\beta I.S_{2}-\delta \beta T-\alpha S_{2}\\ -\mu I+\beta I [S_{1}+S_{2}]-\alpha I+\beta \delta T+\sigma I\\ \mu I-\rho T-\alpha T+\psi T+\varepsilon T\\ -\alpha R +\rho T \end{array}\right\} \end{aligned}$$

### Proof

we create that the inner product of

$$\begin{aligned} T(S_{1(11)}(t)-S_{1(12)}(t),S_{2(21)}(t)-S_{2(22)}(t),I_{31}(t)-I_{32}(t), \end{aligned}$$

$$\begin{aligned} T_{41}(t)-T_{42}(t),R_{51}(t)-R_{52}(t),(v_1,v_2,v_3,v_4,v_5)) \end{aligned}$$

Where

$$\begin{aligned} (S_{1(11)}(t)-S_{1(12)}(t),S_{2(21)}(t)-S_{2(22)}(t),I_{31}(t)-I_{32}(t),T_{41}(t)-T_{42}(t),R_{51}(t)-R_{52}(t)) \end{aligned}$$

are special solutions of the system. We can achieve the following result by using the relationship between inner function and the norm.

$$\begin{aligned} B-\beta ||I_{31}(t)S_{1(11)}(t)-I_{32}(t)S_{1(12)}(t)|| -\delta \beta ||T_{41}(t)-T_{42}(t)|| -\alpha ||S_{1(11)}(t)-S_{1(12)}(t)|| \end{aligned}$$

$$\begin{aligned} \begin{array}{c} \le ||B||||V_1||-\beta ||I_{31}(t)S_{1(11)}(t)-I_{32}(t)S_{1(12)}(t)||||V_1||-\delta \beta ||T_{41}(t)-T_{42}(t)||||V_1||\\ -\alpha ||S_{1(11)}(t)-S_{1(12)}(t)|| ||V_1|| ||V_1||\\ B-\beta ||I_{31}(t)S_{2(21)}(t)-I_{32}(t)S_{2(22)}(t)|| -\delta \beta ||T_{41}(t)-T_{42}(t)|| -\alpha ||S_{2(21)}(t)-S_{2(22)}(t)|| \end{array} \end{aligned}$$

$$\begin{aligned} \begin{array}{c} \le ||B||||V_2||-\beta ||I_{31}(t)S_{2(21)}(t)-I_{32}(t)S_{2(22)}(t)||||V_2||-\delta \beta ||T_{41}(t)-T_{42}(t)||||V_2||\\ -\alpha ||S_{1(11)}(t)-S_{1(12)}(t)|| ||V_2||||V_2||\\ (-\mu || I_{31}(t)-I_{32}(t) ||+\beta || I_{31}(t) (S_{1(11)}(t)+S_{2(21)}(t))- I_{32}(t)(S_{1(12)}(t)+S_{2(22)}(t))||\\ -\alpha ||I_{31}(t)-I_{32}(t)||+\beta \delta ||T_{41}(t)-T_{42}(t)||+||\sigma (t)-\sigma (t)||) \end{array} \end{aligned}$$

$$\begin{aligned} \begin{array}{c} \le -\mu || I_{31}(t)-I_{32}(t) || ||v_3||+\beta || I_{31}(t) (S_{1(11)}(t)+S_{2(21)}(t))- I_{32}(t)(S_{1(12)}(t)+S_{2(22)}(t))|| ||v_3||\\ -\alpha ||I_{31}(t)-I_{32}(t)|| ||v_3||+\beta \delta ||T_{41}(t)-T_{42}(t)|| ||v_3||+||\sigma (t)-\sigma (t)|| ||v_3||| |v_3||\\ ( \mu || I_{31}(t)-I_{32}(t)||-\rho || T_{41}(t)-T_{42}(t)||-\alpha || T_{41}(t)-T_{42}(t)||+\psi ||T_{41}(t)-T_{42}(t)||\\ +\varepsilon ||T_{41}(t)-T_{42}(t)||)\\ \le \mu || I_{31}(t)-I_{32}(t)|| ||v_4||-\rho || T_{41}(t)-T_{42}(t)|| ||v_4||-\alpha || T_{41}(t)-T_{42}(t)|| ||v_4||+\psi ||T_{41}(t)-T_{42}(t)|| ||v_4||\\ +\varepsilon ||T_{41}(t)-T_{42}(t)|| ||v_4|| ||v_4||\\ (- \alpha || R_{51}(t)-R_{52}(t)||+\rho || T_{41}(t)-T_{42}(t)||)\\ \le -\alpha || R_{51}(t)-R_{52}(t)|| ||v_5||+\rho || T_{41}(t)-T_{42}(t)|| ||v_5|| ||v_5|| \end{array} \end{aligned}$$

For large number$( e_1,e_2,e_3,e_4 and e_5)$ these solutions converge to exact solution. Using the concept of topology, we attain for a small parameters $( Xe_1, Xe_2, Xe_3,Xe_4,Xe_5)$

$$\begin{aligned} \Vert S_1(t)-S_{1(11)}(t)||,\Vert S_1(t)-S_{1(12)}(t)<\frac{ Xe_{1}}{\varpi } \end{aligned}$$

$$\begin{aligned} \Vert S_2(t)-S_{2(21)}(t)||,\Vert S_2(t)-S_{2(22)}(t)<\frac{ Xe_{2}}{\xi } \end{aligned}$$

$$\begin{aligned} \Vert I(t)-I_{31}(t)||,\Vert I(t)-I_{32}(t)<\frac{Xe_{3}}{\varphi } \end{aligned}$$

$$\begin{aligned} \Vert T(t)-T_{41}(t)||,\Vert T(t)-T_{42}(t)<\frac{Xe_{4}}{\varsigma } \end{aligned}$$

$$\begin{aligned} \Vert R(t)-R_{51}(t)||,\Vert R(t)-R_{52}(t)<\frac{Xe_{5}}{\lambda } \end{aligned}$$

Where

21 $$\begin{aligned} \varpi =B-\beta ||I_{31}(t)S_{1(11)}(t)-I_{32}(t)S_{1(12)}(t)||-\delta \beta ||T_{41}(t)-T_{42}(t)|| -\alpha ||S_{1(11)}(t)-S_{1(12)}(t)|| \ne 0 \end{aligned}$$

22 $$\begin{aligned} \xi =B-\beta ||I_{31}(t)S_{2(21)}(t)-I_{32}(t)S_{2(22)}(t)||-\delta \beta ||T_{41}(t)-T_{42}(t)|| -\alpha ||S_{2(21)}(t)-S_{2(22)}(t)|| \ne 0 \end{aligned}$$

23 $$\begin{aligned} \varphi =-\mu || I_{31}(t)-I_{32}(t) ||+\beta || I_{31}(t) (S_{1(11)}(t)+S_{2(21)}(t))- I_{32}(t)(S_{1(12)}(t)+S_{2(22)}(t))|| -\alpha ||I_{31}(t)-I_{32}(t)||+\beta \delta ||T_{41}(t)-T_{42}(t)||+||\sigma (t)-\sigma (t)|| \ne 0 \end{aligned}$$

24 $$\begin{aligned} \varsigma = \mu || I_{31}(t)-I_{32}(t)||-\rho || T_{41}(t)-T_{42}(t)||-\alpha || T_{41}(t)-T_{42}(t)||+\psi ||T_{41}(t)-T_{42}(t)|| +\varepsilon ||T_{41}(t)-T_{42}(t)|| \ne 0 \end{aligned}$$

25 $$\begin{aligned} \lambda =- \alpha || R_{51}(t)-R_{52}(t)||+\rho ||T_{41}(t)-T_{42}(t)|| \ne 0, \Vert v_{1}||,\Vert v_{2}||,\Vert v_{3},\Vert v_{4}||,\Vert v_{5}||\ne 0 \end{aligned}$$

Also,

$$\begin{aligned} \begin{array}{c} \Vert (S_{1(11)}(t)-S_{1(12)}(t))||,\Vert (S_{2(21)}(t)-S_{2(22)}(t))||,\Vert (I_{31}(t)-I_{32}(t))||,\\ \Vert (T_{41}(t)-T_{42}(t))||,\Vert (R_{51}(t)-R_{52}(t))|| =0 \end{array} \end{aligned}$$

And,

$$\begin{aligned} S_{1(11)}(t)=S_{1(12)}(t),S_{2(21)}(t)=S_{2(22)}(t),I_{31}(t)=I_{32}(t),T_{41}(t)=T_{42}(t),R_{51}(t)=R_{52}(t). \end{aligned}$$

This complete the proof of uniqueness.

## Solution by ABC techniques

Now by using numerical scheme on the Eqs. (6)-(10). Also by the fundamental theorem of fractional calculus can be used to convert the preceding equation to a fractional integral equation using ABC technique.

26 $$\begin{aligned} S_1(t)-S_1(0)=\frac{\xi }{ABC(1-\xi )}f_{1}(t,K)+\frac{\gamma }{\Gamma (\gamma )\times ABC(\gamma )} \int ^{t}_{0}f_{1}(\eta ,k)(t-\eta )^{\gamma -1}d\eta \end{aligned}$$

27 $$\begin{aligned} S_2(t)-S_2(0)=\frac{\xi }{ABC(1-\xi )}f_{2}(t,K)+\frac{\gamma }{\Gamma (\gamma )\times ABC(\gamma )} \int ^{t}_{0}f_{2}(\eta ,k)(t-\eta )^{\gamma -1}d\eta \end{aligned}$$

28 $$\begin{aligned} I(t)-I(0)=\frac{\xi }{ABC(1-\xi )}f_{3}(t,K)+\frac{\gamma }{\Gamma (\gamma )\times ABC(\gamma )} \int ^{t}_{0}f_{3}(\eta ,k)(t-\eta )^{\gamma -1}d\eta \end{aligned}$$

29 $$\begin{aligned} T(t)-T(0)=\frac{\xi }{ABC(1-\xi )}f_{4}(t,K)+\frac{\gamma }{\Gamma (\gamma )\times ABC(\gamma )} \int ^{t}_{0}f_{4}(\eta , k)(t-\eta )^{\gamma -1}d\eta \end{aligned}$$

30 $$\begin{aligned} R(t)-R(0)=\frac{\xi }{ABC(1-\xi )}f_{5}(t,K)+\frac{\gamma }{\Gamma (\gamma )\times ABC(\gamma )} \int ^{t}_{0}f_{5}(\eta , k)(t-\eta )^{\gamma -1}d\eta \end{aligned}$$

where $$k= S_1(\eta ),S_2(\eta ),I(\eta ),T(\eta ),R(\eta )$$, $$K= S_1,S_2,I,T,R$$ and $$\xi =1-\gamma$$

At $t=t_{n+1}$ and $n=0,1,2,........$, So

31 $$\begin{aligned} S_1(t_{n+1})-S_1(0)=\frac{\xi }{ABC(1-\xi )}f_{1}(t_n,S_1(t_n),S_2(t_n),I(t_n),T(t_n),R(t_n))+\frac{\gamma }{\Gamma (\gamma )\times ABC(\gamma )} \int ^{t_{n+1}}_{0}f_{1}(\eta , k)(t_{n+1}-\eta )^{\gamma -1}d\eta \end{aligned}$$

32 $$\begin{aligned} S_2(t_{n+1})-S_2(0)=\frac{\xi }{ABC(1-\xi )}f_{2}(t_n,S_1(t_n),S_2(t_n),I(t_n),T(t_n),R(t_n))+\frac{\gamma }{\Gamma (\gamma )\times ABC(\gamma )} \int ^{t_{n+1}}_{0}f_{2}(\eta , k)(t_{n+1}-\eta )^{\gamma -1}d\eta \end{aligned}$$

33 $$\begin{aligned} I(t_{n+1})-I(0)=\frac{\xi }{ABC(1-\xi )}f_{3}(t_n,S_1(t_n),S_2(t_n),I(t_n),T(t_n),R(t_n))+\frac{\gamma }{\Gamma (\gamma )\times ABC(\gamma )} \int ^{t_{n+1}}_{0}f_{3}(\eta , k)(t_{n+1}-\eta )^{\gamma -1}d\eta \end{aligned}$$

34 $$\begin{aligned} T(t_{n+1})-T(0)=\frac{\xi }{ABC(1-\xi )}f_{4}(t_n,S_1(t_n),S_2(t_n),I(t_n),T(t_n),R(t_n))+\frac{\gamma }{\Gamma (\gamma )\times ABC(\gamma )} \int ^{t_{n+1}}_{0}f_{4}(\eta ,k)(t_{n+1}-\eta )^{\gamma -1}d\eta \end{aligned}$$

35 $$\begin{aligned} R(t_{n+1})-R(0)=\frac{\xi }{ABC(1-\xi )}f_{5}(t_n,S_1(t_n),S_2(t_n),I(t_n),T(t_n),R(t_n))+\frac{\gamma }{\Gamma (\gamma )\times ABC(\gamma )} \int ^{t_{n+1}}_{0}f_{5}(\eta , k)(t_{n+1}-\eta )^{\gamma -1}d\eta \end{aligned}$$

36 $$\begin{aligned} S_1(t_{n+1})-S_1(0)=\frac{\xi }{ABC(1-\xi )}f_{1}(t_n,S_1(t_n),S_2(t_n),I(t_n),T(t_n),R(t_n))+\frac{\gamma }{\Gamma (\gamma )\times ABC(\gamma )} \sum \limits ^{n}_{k=0}\int ^{t_{n+1}}_{0}f_{1}(\eta , k)(t_{n+1}-\eta )^{\gamma -1}d\eta \end{aligned}$$

37 $$\begin{aligned} S_2(t_{n+1})-S_2(0)=\frac{\xi }{ABC(1-\xi )}f_{2}(t_n,S_1(t_n),S_2(t_n),I(t_n),T(t_n),R(t_n))+\frac{\gamma }{\Gamma (\gamma )\times ABC(\gamma )} \sum \limits ^{n}_{k=0}\int ^{t_{n+1}}_{0}f_{2}(\eta , k)(t_{n+1}-\eta )^{\gamma -1}d\eta \end{aligned}$$

38 $$\begin{aligned} I(t_{n+1})-I(0)=\frac{\xi }{ABC(1-\xi )}f_{3}(t_n,S_1(t_n),S_2(t_n),I(t_n),T(t_n),R(t_n))+\frac{\gamma }{\Gamma (\gamma )\times ABC(\gamma )} \sum \limits ^{n}_{k=0}\int ^{t_{n+1}}_{0}f_{3}(\eta , k)(t_{n+1}-\eta )^{\gamma -1}d\eta \end{aligned}$$

39 $$\begin{aligned} T(t_{n+1})-T(0)=\frac{\xi }{ABC(1-\xi )}f_{4}(t_n,S_1(t_n),S_2(t_n),I(t_n),T(t_n),R(t_n))+\frac{\gamma }{\Gamma (\gamma )\times ABC(\gamma )} \sum \limits ^{n}_{k=0}\int ^{t_{n+1}}_{0}f_{4}(\eta , k)(t_{n+1}-\eta )^{\gamma -1}d\eta \end{aligned}$$

40 $$\begin{aligned} R(t_{n+1})-R(0)=\frac{\xi }{ABC(1-\xi )}f_{5}(t_n,S_1(t_n),S_2(t_n),I(t_n),T(t_n),R(t_n))+\frac{\gamma }{\Gamma (\gamma )\times ABC(\gamma )} \sum \limits ^{n}_{k=0}\int ^{t_{n+1}}_{0}f_{5}(\eta , k)(t_{n+1}-\eta )^{\gamma -1}d\eta \end{aligned}$$

Applying two-step Lagrange polynomial interpolation , under the interval $[t_{j},t_{j+1}]$ ,can be approximated as follows:

41 $$\begin{aligned} \begin{array}{c} S_1(t_{n+1})=S_1(0)+\frac{\xi }{ABC(1-\xi )}f_{1}(t_{n},S_1(t_{n}),S_2(t_{n}),I(t_{n}),T(t_{n}),R(t_{n}))\\ +\frac{1-\xi }{\Gamma (1-\xi )\times ABC(1-\xi )}\sum \limits ^{n}_{j=0}\bigg (\frac{f_{1}(t_{j}-S_{1(j)}-S_{2(j)}-I_{j}-T_{j}-R_{j})}{h}\int ^{t_{j+1}}_{t_{j}}(\eta -t_{j-1})(t_{n+1}-\eta )^{\gamma -1}d\eta \\ -\frac{f_{1}(t_{j-1},S_{1(j-1)},S_{2(j-1)},I_{j-1},T_{j-1},R_{j-1})}{h}\int ^{t_{j+1}}_{t_{j}}(\eta -t_{j})(t_{n+1}-\eta )^{\gamma -1}d\eta \bigg ) \end{array} \end{aligned}$$

42 $$\begin{aligned} \begin{array}{c} S_2(t_{n+1})=S_2(0)+\frac{\xi }{ABC(1-\xi )}f_{2}(t_{n},S_1(t_{n}),S_2(t_{n}),I(t_{n}),T(t_{n}),R(t_{n}))\\ +\frac{1-\xi }{\Gamma (1-\xi )\times ABC(1-\xi )}\sum \limits ^{n}_{j=0}\bigg (\frac{f_{2}(t_{j}-S_{1(j)}-S_{2(j)}-I_{j}-T_{j}-R_{j})}{h}\int ^{t_{j+1}}_{t_{j}}(\eta -t_{j-1})(t_{n+1}-\eta )^{\gamma -1}d\eta \\ -\frac{f_{2}(t_{j-1},S_{1(j-1)},S_{2(j-1)},I_{j-1},T_{j-1},R_{j-1})}{h}\int ^{t_{j+1}}_{t_{j}}(\eta -t_{j})(t_{n+1}-\eta )^{\gamma -1}d\eta \bigg ) \end{array} \end{aligned}$$

43 $$\begin{aligned} \begin{array}{c} I(t_{n+1})=I(0)+\frac{\xi }{ABC(1-\xi )}f_{3}(t_{n},S_1(t_{n}),S_2(t_{n}),I(t_{n}),T(t_{n}),R(t_{n}))\\ +\frac{1-\xi }{\Gamma (1-\xi )\times ABC(1-\xi )}\sum \limits ^{n}_{j=0}\bigg (\frac{f_{3}(t_{j}-S_{1(j)}-S_{2(j)}-I_{j}-T_{j}-R_{j})}{h}\int ^{t_{j+1}}_{t_{j}}(\eta -t_{j-1})(t_{n+1}-\eta )^{\gamma -1}d\eta \\ -\frac{f_{3}(t_{j-1},S_{1(j-1)},S_{2(j-1)},I_{j-1},T_{j-1},R_{j-1})}{h}\int ^{t_{j+1}}_{t_{j}}(\eta -t_{j})(t_{n+1}-\eta )^{\gamma -1}d\eta \bigg ) \end{array} \end{aligned}$$

44 $$\begin{aligned} \begin{array}{c} T(t_{n+1})=T(0)+\frac{\xi }{ABC(1-\xi )}f_{4}(t_{n},S_1(t_{n}),S_2(t_{n}),I(t_{n}),T(t_{n}),R(t_{n}))\\ +\frac{1-\xi }{\Gamma (1-\xi )\times ABC(1-\xi )}\sum \limits ^{n}_{j=0}\bigg (\frac{f_{4}(t_{j}-S_{1(j)}-S_{2(j)}-I_{j}-T_{j}-R_{j})}{h}\int ^{t_{j+1}}_{t_{j}}(\eta -t_{j-1})(t_{n+1}-\eta )^{\gamma -1}d\eta \\ -\frac{f_{4}(t_{j-1},S_{1(j-1)},S_{2(j-1)},I_{j-1},T_{j-1},R_{j-1})}{h}\int ^{t_{j+1}}_{t_{j}}(\eta -t_{j})(t_{n+1}-\eta )^{\gamma -1}d\eta \bigg ) \end{array} \end{aligned}$$

45 $$\begin{aligned} \begin{array}{c} R(t_{n+1})=R(0)+\frac{\xi }{ABC(1-\xi )}f_{5}(t_{n},S_1(t_{n}),S_2(t_{n}),I(t_{n}),T(t_{n}),R(t_{n}))\\ +\frac{1-\xi }{\Gamma (1-\xi )\times ABC(1-\xi )}\sum \limits ^{n}_{j=0}\bigg (\frac{f_{5}(t_{j}-S_{1(j)}-S_{2(j)}-I_{j}-T_{j}-R_{j})}{h}\int ^{t_{j+1}}_{t_{j}}(\eta -t_{j-1})(t_{n+1}-\eta )^{\gamma -1}d\eta \\ -\frac{f_{5}(t_{j-1},S_{1(j-1)},S_{2(j-1)},I_{j-1},T_{j-1},R_{j-1})}{h}\int ^{t_{j+1}}_{t_{j}}(\eta -t_{j})(t_{n+1}-\eta )^{\gamma -1}d\eta \bigg ) \end{array} \end{aligned}$$

For simplicity we let $A_{\gamma ,j,1}$ and $A_{\gamma ,j,2}$ from Eqs. (41)-(45)

46 $$\begin{aligned} A_{\gamma ,j,1}=\int ^{t_{j+1}}_{t_{j}}(\eta -t_{j-1})(t_{n+1}-\eta )^{\gamma -1}d\eta \end{aligned}$$

47 $$\begin{aligned} A_{\gamma ,j,2}=\int ^{t_{j+1}}_{t_{j}}(\eta -t_{j})(t_{n+1}-\eta )^{\gamma -1}d\eta \end{aligned}$$

Integrating the Eqs. (46) and (47)

48 $$\begin{aligned} A_{\gamma ,j,1}=h^{2-\xi }\frac{Q}{(1-\xi )(2-\xi )} \end{aligned}$$

49 $$\begin{aligned} A_{\gamma ,j,2}=h^{2-\xi }\frac{r}{(1-\xi )(2-\xi )} \end{aligned}$$

where $$Q=(n+1-j)^{1-\xi }(n-j+3-\xi )-(n-j)^{1-\xi }(n-j+2+2(1-\xi )), r=(n+1-j)^{2-\xi }-(n-j)^{1-\xi }(n-j+2-\xi )$$

In the next section, we’ll look at the numerical error of the above estimate.

## Error analysis

In this session, we’ll figure out what went wrong when we used our method to estimate the fractional partial differential equation.

### Theorem 3

The system of equation must be a fractional derivative with non-local and non-singular kernel. As a result, the function’s second derivative is bounded, and the error is calculated to fulfil.

$$\begin{aligned} |R^{\gamma }_{n}|\le \frac{\gamma (h^{\gamma +2})}{2ABC(\gamma )\Gamma (\gamma +2)}\max _{[0,t_{n+1}]}|\frac{\partial ^{2}}{\partial \eta ^{2}}|f_{1}(\eta ,k)|\times ((n+1)^{n}-\gamma (n^{\gamma }))\frac{n(n+4+2\gamma )}{2} \end{aligned}$$

$$\begin{aligned} |R^{\gamma }_{n}|\le \frac{\gamma (h^{\gamma +2})}{2ABC(\gamma )\Gamma (\gamma +2)}\max _{[0,t_{n+1}]}|\frac{\partial ^{2}}{\partial \eta ^{2}}|f_{2}(\eta ,S_1(\eta ),k)|\times ((n+1)^{n}-\gamma (n^{\gamma }))\frac{n(n+4+2\gamma )}{2} \end{aligned}$$

$$\begin{aligned} |R^{\gamma }_{n}|\le \frac{\gamma (h^{\gamma +2})}{2ABC(\gamma )\Gamma (\gamma +2)}\max _{[0,t_{n+1}]}|\frac{\partial ^{2}}{\partial \eta ^{2}}|f_{3}(\eta ,k)|\times ((n+1)^{n}-\gamma (n^{\gamma }))\frac{n(n+4+2\gamma )}{2} \end{aligned}$$

$$\begin{aligned} |R^{\gamma }_{n}|\le \frac{\gamma (h^{\gamma +2})}{2ABC(\gamma )\Gamma (\gamma +2)}\max _{[0,t_{n+1}]}|\frac{\partial ^{2}}{\partial \eta ^{2}}|f_{4}(\eta ,k)|\times ((n+1)^{n}-\gamma (n^{\gamma }))\frac{n(n+4+2\gamma )}{2} \end{aligned}$$

$$\begin{aligned} |R^{\gamma }_{n}|\le \frac{\gamma (h^{\gamma +2})}{2ABC(\gamma )\Gamma (\gamma +2)}\max _{[0,t_{n+1}]}|\frac{\partial ^{2}}{\partial \eta ^{2}}|f_{5}(\eta ,k)|\times ((n+1)^{n}-\gamma (n^{\gamma }))\frac{n(n+4+2\gamma )}{2} \end{aligned}$$

### Proof

we consider the model given in Eqs. (6)-(10) to develops a numerical algorithm as follows.

50 $$\begin{aligned} S_1(t_{n+1})-S_1(0)=\frac{1-\gamma }{ABC(\gamma )}f_{1}(t_n,S_1(t_n),S_2(t_n),I(t_n),T(t_n),R(t_n))+\frac{\gamma }{\Gamma (\gamma )\times ABC(\gamma )}\int ^{t_{n+1}}_{0}f_{1}(\eta ,k)(t_{n+1}-\eta )^{\gamma -1}d\eta \end{aligned}$$

51 $$\begin{aligned} S_2(t_{n+1})-S_2(0)=\frac{1-\gamma }{ABC(\gamma )}f_{2}(t_n,S_1(t_n),S_2(t_n),I(t_n),T(t_n),R(t_n))+\frac{\gamma }{\Gamma (\gamma )\times ABC(\gamma )}\int ^{t_{n+1}}_{0}f_{2}(\eta ,k)(t_{n+1}-\eta )^{\gamma -1}d\eta \end{aligned}$$

52 $$\begin{aligned} I(t_{n+1})-I(0)=\frac{1-\gamma }{ABC(\gamma )}f_{3}(t_n,S_1(t_n),S_2(t_n),I(t_n),T(t_n),R(t_n))+\frac{\gamma }{\Gamma (\gamma )\times ABC(\gamma )}\int ^{t_{n+1}}_{0}f_{3}(\eta ,k)(t_{n+1}-\eta )^{\gamma -1}d\eta \end{aligned}$$

53 $$\begin{aligned} T(t_{n+1})-T(0)=\frac{1-\gamma }{ABC(\gamma )}f_{4}(t_n,S_1(t_n),S_2(t_n),I(t_n),T(t_n),R(t_n))+\frac{\gamma }{\Gamma (\gamma )\times ABC(\gamma )}\int ^{t_{n+1}}_{0}f_{4}(\eta ,k)(t_{n+1}-\eta )^{\gamma -1}d\eta \end{aligned}$$

54 $$\begin{aligned} R(t_{n+1})-R(0)=\frac{1-\gamma }{ABC(\gamma )}f_{5}(t_n,S_1(t_n),S_2(t_n),I(t_n),T(t_n),R(t_n))+\frac{\gamma }{\Gamma (\gamma )\times ABC(\gamma )} \int ^{t_{n+1}}_{0}f_{5}(\eta ,k)(t_{n+1}-\eta )^{\gamma -1}d\eta \end{aligned}$$

55 $$\begin{aligned} S_1(t_{n+1})-S_1(0)=\frac{1-\gamma }{ABC(\gamma )}f_{1}(t_n,S_1(t_n),S_2(t_n),I(t_n),T(t_n),R(t_n))+\frac{\gamma }{\Gamma (\gamma )\times ABC(\gamma )}\sum \limits ^{n}_{j=0}\int ^{t_{n+1}}_{0}f_{1}(\eta ,k)(t_{n+1}-\eta )^{\gamma -1}d\eta \end{aligned}$$

56 $$\begin{aligned} S_2(t_{n+1})-S_2(0)=\frac{1-\gamma }{ABC(\gamma )}f_{2}(t_n,S_1(t_n),S_2(t_n),I(t_n),T(t_n),R(t_n))+\frac{\gamma }{\Gamma (\gamma )\times ABC(\gamma )}\sum \limits ^{n}_{j=0}\int ^{t_{n+1}}_{0}f_{2}(\eta ,k)(t_{n+1}-\eta )^{\gamma -1}d\eta \end{aligned}$$

57 $$\begin{aligned} I(t_{n+1})-I(0)=\frac{1-\gamma }{ABC(\gamma )}f_{3}(t_n,S_1(t_n),S_2(t_n),I(t_n),T(t_n),R(t_n))+\frac{\gamma }{\Gamma (\gamma )\times ABC(\gamma )}\sum \limits ^{n}_{j=0}\int ^{t_{n+1}}_{0}f_{3}(\eta ,k)(t_{n+1}-\eta )^{\gamma -1}d\eta \end{aligned}$$

58 $$\begin{aligned} T(t_{n+1})-T(0)=\frac{1-\gamma }{ABC(\gamma )}f_{4}(t_n,S_1(t_n),S_2(t_n),I(t_n),T(t_n),R(t_n))+\frac{\gamma }{\Gamma (\gamma )\times ABC(\gamma )}\sum \limits ^{n}_{j=0}\int ^{t_{n+1}}_{0}f_{4}(\eta ,k)(t_{n+1}-\eta )^{\gamma -1}d\eta \end{aligned}$$

59 $$\begin{aligned} R(t_{n+1})-R(0)=\frac{1-\gamma }{ABC(\gamma )}f_{5}(t_n,S_1(t_n),S_2(t_n),I(t_n),T(t_n),R(t_n))+\frac{\gamma }{\Gamma (\gamma )\times ABC(\gamma )}\sum \limits ^{n}_{j=0}\int ^{t_{n+1}}_{0}f_{5}(\eta ,k)(t_{n+1}-\eta )^{\gamma -1}d\eta \end{aligned}$$

For the function $f(\eta ,S_1(\eta ),S_2(\eta ),I(\eta ),T(\eta ),R(\eta )$ we using the Lagrange polynomial interpolation. Also it is unquestionably true that the function $\eta \rightarrow ^{yield}(\eta -t_{j-1})(t_{n+1}-\eta )^{-\xi }$is positive within the interval$[t_{j},t_{j+1}]$, therefore there exist $\xi _{j}\epsilon [t_{j},t_{j+1}]$, such that, we can write its simplified form as

60 $$\begin{aligned} R^{\gamma _{1}}_{n}=\frac{1-\xi }{ABC(1-\xi )\Gamma (1-\xi )}\times \sum \limits ^{n}_{j=0}\frac{\partial ^{2}}{\partial \eta ^{2}}|f_{1}(\eta ,k)|_{\eta =\xi _{\eta }}\frac{\xi _{j}-t_{j}}{2}\int ^{t_{j+1}}_{t_{j}}(\eta -t_{j-1})(t_{n+1}-\eta )^{\gamma -1}d\eta =\frac{1-\xi }{ABC(1-\xi )\Gamma (1-\xi )}\sum \limits ^{n}_{j=0}\frac{\partial ^{2}}{\partial \eta ^{2}}|f_{1}(\eta ,k)|_{\eta =\xi _{\eta }}\frac{\xi _{j}-t_{j}}{2}\times (A_{\gamma ,j,1})h^{\gamma +1} \end{aligned}$$

61 $$\begin{aligned} R^{\gamma _{2}}_{n}=\frac{1-\xi }{ABC(1-\xi )\Gamma (1-\xi )}\times \sum \limits ^{n}_{j=0}\frac{\partial ^{2}}{\partial \eta ^{2}}|f_{2}(\eta ,k)|_{\eta =\xi _{\eta }}\frac{\xi _{j}-t_{j}}{2}\int ^{t_{j+1}}_{t_{j}}(\eta -t_{j-1})(t_{n+1}-\eta )^{\gamma -1}d\eta =\frac{1-\xi }{ABC(1-\xi )\Gamma (1-\xi )}\sum \limits ^{n}_{j=0}\frac{\partial ^{2}}{\partial \eta ^{2}}|f_{2}(\eta ,k)|_{\eta =\xi _{\eta }}\frac{\xi _{j}-t_{j}}{2}\times (A_{\gamma ,j,1})h^{\gamma +1} \end{aligned}$$

62 $$\begin{aligned} R^{\gamma _{3}}_{n}=\frac{1-\xi }{ABC(1-\xi )\Gamma (1-\xi )}\times \sum \limits ^{n}_{j=0}\frac{\partial ^{2}}{\partial \eta ^{2}}|f_{3}(\eta ,k)|_{\eta =\xi _{\eta }}\frac{\xi _{j}-t_{j}}{2}\int ^{t_{j+1}}_{t_{j}}(\eta -t_{j-1})(t_{n+1}-\eta )^{\gamma -1}d\eta =\frac{1-\xi }{ABC(1-\xi )\Gamma (1-\xi )}\sum \limits ^{n}_{j=0}\frac{\partial ^{2}}{\partial \eta ^{2}}|f_{3}(\eta ,k)|_{\eta =\xi _{\eta }}\frac{\xi _{j}-t_{j}}{2}\times (A_{\gamma ,j,1})h^{\gamma +1} \end{aligned}$$

63 $$\begin{aligned} R^{\gamma _{4}}_{n}=\frac{1-\xi }{ABC(1-\xi )\Gamma (1-\xi )}\times \sum \limits ^{n}_{j=0}\frac{\partial ^{2}}{\partial \eta ^{2}}|f_{4}(\eta ,k)|_{\eta =\xi _{\eta }}\frac{\xi _{j}-t_{j}}{2}\int ^{t_{j+1}}_{t_{j}}(\eta -t_{j-1})(t_{n+1}-\eta )^{\gamma -1}d\eta =\frac{1-\xi }{ABC(1-\xi )\Gamma (1-\xi )}\sum \limits ^{n}_{j=0}\frac{\partial ^{2}}{\partial \eta ^{2}}|f_{4}(\eta ,k)|_{\eta =\xi _{\eta }}\frac{\xi _{j}-t_{j}}{2}\times (A_{\gamma ,j,1})h^{\gamma +1} \end{aligned}$$

64 $$\begin{aligned} R^{\gamma _{5}}_{n}=\frac{1-\xi }{ABC(1-\xi )\Gamma (1-\xi )}\times \sum \limits ^{n}_{j=0}\frac{\partial ^{2}}{\partial \eta ^{2}}|f_{5}(\eta ,k)|_{\eta =\xi _{\eta }}\frac{\xi _{j}-t_{j}}{2}\int ^{t_{j+1}}_{t_{j}}(\eta -t_{j-1})(t_{n+1}-\eta )^{\gamma -1}d\eta =\frac{1-\xi }{ABC(1-\xi )\Gamma (1-\xi )}\sum \limits ^{n}_{j=0}\frac{\partial ^{2}}{\partial \eta ^{2}}|f_{5}(\eta ,k)|_{\eta =\xi _{\eta }}\frac{\xi _{j}-t_{j}}{2}\times (A_{\gamma ,j,1})h^{\gamma +1} \end{aligned}$$

where

65 $$\begin{aligned} A_{1-\xi ,j,1}=\frac{(n+1-j)^{1-\xi }(n-j+3-\xi )-(n-j)^{1-\xi }(n-j+2+2(1-\xi ))}{(1-\xi )(2-\xi } \end{aligned}$$

Now put the value of $A_{1-\xi ,j,1}$ and applying the norm on both sides of the equation and making use of the norm properties. Now,

66 $$\begin{aligned} |R^{\gamma _{1}}_{n}|\le \frac{(1-\xi )(h)^{3-\xi }}{2ABC(1-\xi )\Gamma (3-\xi )}\max _{[0,t_{n+1}]}|\frac{\partial ^{2}}{\partial \eta ^{2}}|f_{1}(\eta ,k)||\sum \limits ^{n}_{j=0}(Q)| \end{aligned}$$

67 $$\begin{aligned} |R^{\gamma _{2}}_{n}|\le \frac{(1-\xi )(h)^{3-\xi }}{2ABC(1-\xi )\Gamma (3-\xi )}\max _{[0,t_{n+1}]}|\frac{\partial ^{2}}{\partial \eta ^{2}}|f_{2}(\eta ,k)||\sum \limits ^{n}_{j=0}(Q)| \end{aligned}$$

68 $$\begin{aligned} |R^{\gamma _{3}}_{n}|\le \frac{(1-\xi )(h)^{3-\xi }}{2ABC(1-\xi )\Gamma (3-\xi )}\max _{[0,t_{n+1}]}|\frac{\partial ^{2}}{\partial \eta ^{2}}|f_{3}(\eta ,k)||\sum \limits ^{n}_{j=0}(Q)| \end{aligned}$$

69 $$\begin{aligned} |R^{\gamma _{4}}_{n}|\le \frac{(1-\xi )(h)^{3-\xi }}{2ABC(1-\xi )\Gamma (3-\xi )}\max _{[0,t_{n+1}]}|\frac{\partial ^{2}}{\partial \eta ^{2}}|f_{4}(\eta ,k)||\sum \limits ^{n}_{j=0}(Q)| \end{aligned}$$

70 $$\begin{aligned} |R^{\gamma _{5}}_{n}|\le \frac{(1-\xi )(h)^{3-\xi }}{2ABC(1-\xi )\Gamma (3-\xi )}\max _{[0,t_{n+1}]}|\frac{\partial ^{2}}{\partial \eta ^{2}}|f_{5}(\eta ,k)||\sum \limits ^{n}_{j=0}(Q)| \end{aligned}$$

The summation of the right-hand side of the above equation converge as follow

$$\begin{aligned} \begin{array}{c} ((n+1-j)^{1-\xi }(n-j+3-\xi )-(n-j)^{1-\xi }(n-j+2+2(1-\xi )))\\ =((n+1-j)^{1-\xi }(n-j+3-\xi )-(n-j)^{1-\xi }(n-j+2+(1-\xi )+(1-\xi ))\\ =((n-j+3-\xi )((n+1-j)^{1-\xi }-(n-j)^{1-\xi }(1-\xi ))\\ (n+1-j)^{1-\xi }-(1-\xi )(n-j)^{1-\xi }\le ((n+1)^{1-\xi }-(1-\xi )(n)^{1-\xi }) \end{array} \end{aligned}$$

71 $$\begin{aligned} \sum \limits ^{n}_{j=0}(n-j+2+\rho )=\frac{n(n+4+2\rho )}{2} \end{aligned}$$

Thus

72 $$\begin{aligned} |R^{\gamma _{1}}_{n}|\le \frac{(1-\xi )(h)^{3-\xi }}{2ABC(1-\xi )\Gamma (3-\xi )}\max _{[0,t_{n+1}]}|\frac{\partial ^{2}}{\partial \eta ^{2}}|f_{1}(\eta ,k)|((n+1)^{\gamma }-\gamma (n)^{\gamma })\frac{n(n+4+2\gamma )}{2} \end{aligned}$$

73 $$\begin{aligned} |R^{\gamma _{2}}_{n}|\le \frac{(1-\xi )(h)^{3-\xi }}{2ABC(1-\xi )\Gamma (3-\xi )}\max _{[0,t_{n+1}]}|\frac{\partial ^{2}}{\partial \eta ^{2}}|f_{2}(\eta ,k)|((n+1)^{\gamma }-\gamma (n)^{\gamma })\frac{n(n+4+2\gamma )}{2} \end{aligned}$$

74 $$\begin{aligned} |R^{\gamma _{3}}_{n}|\le \frac{(1-\xi )(h)^{3-\xi }}{2ABC(1-\xi )\Gamma (3-\xi )}\max _{[0,t_{n+1}]}|\frac{\partial ^{2}}{\partial \eta ^{2}}|f_{3}(\eta ,k)|((n+1)^{\gamma }-\gamma (n)^{\gamma })\frac{n(n+4+2\gamma )}{2} \end{aligned}$$

75 $$\begin{aligned} |R^{\gamma _{4}}_{n}|\le \frac{(1-\xi )(h)^{3-\xi }}{2ABC(1-\xi )\Gamma (3-\xi )}\max _{[0,t_{n+1}]}|\frac{\partial ^{2}}{\partial \eta ^{2}}|f_{4}(\eta ,k)|((n+1)^{\gamma }-\gamma (n)^{\gamma })\frac{n(n+4+2\gamma )}{2} \end{aligned}$$

76 $$\begin{aligned} |R^{\gamma _{5}}_{n}|\le \frac{(1-\xi )(h)^{3-\xi }}{2ABC(1-\xi )\Gamma (3-\xi )}\max _{[0,t_{n+1}]}|\frac{\partial ^{2}}{\partial \eta ^{2}}|f_{5}(\eta ,k)|((n+1)^{\gamma }-\gamma (n)^{\gamma })\frac{n(n+4+2\gamma )}{2} \end{aligned}$$

This complete the proof.

## Numerical scheme by fractal fractional of Hybrid NAR-RBFs networks for Covid-19 model :

We can extend the fractional order model by using the fractal fractional operators with mittag-leffler kernel. Mathematical model for COVID-19 in fractal fractional operators with mittag-leffler kernel define is as follows.

77 $$\begin{aligned} ^{FFM}_{0}D^{\gamma ,\phi }_{t} S_{1}=B-\beta I\times S_{1}-\delta \times \beta \times T-\alpha \times S{1} \end{aligned}$$

78 $$\begin{aligned} ^{FFM}_{0}D^{\gamma ,\phi }_{t}S{2}=B-\beta \times I\times S_{2} -\delta \times \beta \times T-\alpha S_{2} \end{aligned}$$

79 $$\begin{aligned} ^{FFM}_{0}D^{\gamma ,\phi }_{t}I=- \mu \times I+\beta \times I\times [S_{1}+S_{2}]-\alpha \times I+\beta \delta \times T+\sigma \times I \end{aligned}$$

80 $$\begin{aligned} ^{FFM}_{0}D^{\gamma ,\phi }_{t}T=\mu \times I-\rho \times T-\alpha \times T+\psi \times T+\varepsilon \times T \end{aligned}$$

81 $$\begin{aligned} ^{FFM}_{0}D^{\gamma ,\phi }_{t}R=- \alpha R+\rho T \end{aligned}$$

Here $^{FFM}_{0}D^{\gamma ,\phi }_{t}$ shows the fractal fractional operator with mittag-leffler kernel, and $0< \gamma ,\phi \le 1$. Initial conditions of this system’s is: $S_{1}(0)=S_{1(0)}$ , $S_2(0)=S_{2(0)}$ , $I(0)=I_{0}$ , $T(0)=T_0$ , $R(0)=R_0$

Numerical schemes for this model using Lagrangian piece wise interpolation under considering the fractal fractional operators with mittag-leffler kernel in this part. The designed technique is based on the numerical method of Adams-Bashforth.

82 $$\begin{aligned} S_1(t)=S(0)+\frac{\phi t^{\phi -1}(1-\gamma )}{AB(\gamma )}g_{1}(t,S_1,S_2,I,T,R)+\frac{\gamma \phi }{AB(\gamma )\Gamma (\gamma )}\int ^{t}_{0}\Psi ^{\phi -1}(t-\Psi )g_{1}(\Psi ,S_1,S_2,I,T,R)d\Psi \end{aligned}$$

83 $$\begin{aligned} S_2(t)=S(0)+\frac{\phi t^{\phi -1}(1-\gamma )}{AB(\gamma )}g_{2}(t,S_1,S_2,I,T,R)+\frac{\gamma \phi }{AB(\gamma )\Gamma (\gamma )}\int ^{t}_{0}\Psi ^{\phi -1}(t-\Psi )g_{1}(\Psi ,S_1,S_2,I,T,R)d\Psi \end{aligned}$$

84 $$\begin{aligned} I(t)=I(0)+\frac{\phi t^{\phi -1}(1-\gamma )}{AB(\gamma )}g_{3}(t,S_1,S_2,I,T,R)+\frac{\gamma \phi }{AB(\gamma )\Gamma (\gamma )}\int ^{t}_{0}\Psi ^{\phi -1}(t-\Psi )g_{1}(\Psi ,S_1,S_2,I,T,R)d\Psi \end{aligned}$$

85 $$\begin{aligned} T(t)=T(0)+\frac{\phi t^{\phi -1}(1-\gamma )}{AB(\gamma )}g_{4}(t,S_1,S_2,I,T,R)+\frac{\gamma \phi }{AB(\gamma )\Gamma (\gamma )}\int ^{t}_{0}\Psi ^{\phi -1}(t-\Psi )g_{1}(\Psi ,S_1,S_2,I,T,R)d\Psi \end{aligned}$$

86 $$\begin{aligned} R(t)=R(0)+\frac{\phi t^{\phi -1}(1-\gamma )}{AB(\gamma )}g_{5}(t,S_1,S_2,I,T,R)+\frac{\gamma \phi }{AB(\gamma )\Gamma (\gamma )}\int ^{t}_{0}\Psi ^{\phi -1}(t-\Psi )g_{1}(\Psi ,S_1,S_2,I,T,R)d\Psi \end{aligned}$$

At $$t = t_{b+1}$$, So

87 $$\begin{aligned} S_1^{b+1}=S_1^{0}+\frac{\phi t^{\phi -1}(1-\gamma )}{AB(\gamma )}g_{1}(t_{b},S_1^{b},S_2^{b},I^{b},T^{b},R^{b})+\frac{\gamma \phi }{AB(\gamma )\Gamma (\gamma )}\int ^{t}_{0}\psi ^{\phi -1}(t-\psi )g_{1}(\psi ,S_1,S_2,I,T,R)d\psi \end{aligned}$$

88 $$\begin{aligned} S_2^{b+1}=S_2^{0}+\frac{\phi t^{\phi -1}(1-\gamma )}{AB(\gamma )}g_{2}(t_{b},S_1^{b},S_2^{b},I^{b},T^{b},R^{b})+\frac{\gamma \phi }{AB(\gamma )\Gamma (\gamma )}\int ^{t}_{0}\psi ^{\phi -1}(t-\psi )g_{2}(\psi ,S_1,S_2,I,T,R)d\psi \end{aligned}$$

89 $$\begin{aligned} I^{b+1}=I^{0}+\frac{\phi t^{\phi -1}(1-\gamma )}{AB(\gamma )}g_{3}(t_{b},S_1^{b},S_2^{b},I^{b},T^{b},R^{b})+\frac{\gamma \phi }{AB(\gamma )\Gamma (\gamma )}\int ^{t}_{0}\psi ^{\phi -1}(t-\psi )g_{3}(\psi ,S_1,S_2,I,T,R)d\psi \end{aligned}$$

90 $$\begin{aligned} T^{b+1}=T^{0}+\frac{\phi t^{\phi -1}(1-\gamma )}{AB(\gamma )}g_{4}(t_{b},S_1^{b},S_2^{b},I^{b},T^{b},R^{b})+\frac{\gamma \phi }{AB(\gamma )\Gamma (\gamma )}\int ^{t}_{0}\psi ^{\phi -1}(t-\psi )g_{4}(\psi ,S_1,S_2,I,T,R)d\psi \end{aligned}$$

91 $$\begin{aligned} R^{b+1}=R^{0}+\frac{\phi t^{\phi -1}(1-\gamma )}{AB(\gamma )}g_{5}(t_{b},S_1^{b},S_2^{b},I^{b},T^{b},R^{b})+\frac{\gamma \phi }{AB(\gamma )\Gamma (\gamma )}\int ^{t}_{0}\psi ^{\phi -1}(t-\psi )g_{5}(\psi ,S_1,S_2,I,T,R)d\psi \end{aligned}$$

We get the following result by approximating the R.H.S of the integrals ,

92 $$\begin{aligned} S_1^{b+1}=S_1^{0}+\frac{\phi t^{\phi -1}_{b}(1-\gamma )}{AB(\gamma )}g_{1}(t_{b},S_1^{b},S_2^{b},I^{b},T^{b},R^{b})+\frac{\gamma \phi }{AB(\gamma )\Gamma (\gamma )}\sum \limits ^{b}_{c=0}\int ^{t_{g+1}}_{t_{g}}\psi ^{\phi -1}(t_{b+1}-\psi )g_{1}(\psi ,S_1,S_2,I,T,R)d\psi \end{aligned}$$

93 $$\begin{aligned} S_2^{b+1}=S_2^{0}+\frac{\phi t^{\phi -1}_{b}(1-\gamma )}{AB(\gamma )}g_{2}(t_{b},S_1^{b},S_2^{b},I^{b},T^{b},R^{b})+\frac{\gamma \phi }{AB(\gamma )\Gamma (\gamma )}\sum \limits ^{b}_{c=0}\int ^{t_{g+1}}_{t_{g}}\psi ^{\phi -1}(t_{b+1}-\psi )g_{2}(\psi ,S_1,S_2,I,T,R)d\psi \end{aligned}$$

94 $$\begin{aligned} I^{b+1}=I^{0}+\frac{\phi t^{\phi -1}_{b}(1-\gamma )}{AB(\gamma )}g_{3}(t_{b},S_1^{b},S_2^{b},I^{b},T^{b},R^{b})+\frac{\gamma \phi }{AB(\gamma )\Gamma (\gamma )}\sum \limits ^{b}_{c=0}\int ^{t_{g+1}}_{t_{g}}\psi ^{\phi -1}(t_{b+1}-\psi )g_{3}(\psi ,S_1,S_2,I,T,R)d\psi \end{aligned}$$

95 $$\begin{aligned} T^{b+1}=T^{0}+\frac{\phi t^{\phi -1}_{b}(1-\gamma )}{AB(\gamma )}g_{4}(t_{b},S_1^{b},S_2^{b},I^{b},T^{b},R^{b})+\frac{\gamma \phi }{AB(\gamma )\Gamma (\gamma )}\sum \limits ^{b}_{c=0}\int ^{t_{g+1}}_{t_{g}}\psi ^{\phi -1}(t_{b+1}-\psi )g_{4}(\psi ,S_1,S_2,I,T,R)d\psi \end{aligned}$$

96 $$\begin{aligned} R^{b+1}=R^{0}+\frac{\phi t^{\phi -1}_{b}(1-\gamma )}{AB(\gamma )}g_{5}(t_{b},S_1^{b},S_2^{b},I^{b},T^{b},R^{b})+\frac{\gamma \phi }{AB(\gamma )\Gamma (\gamma )}\sum \limits ^{b}_{c=0}\int ^{t_{g+1}}_{t_{g}}\psi ^{\phi -1}(t_{b+1}-\psi )g_{5}(\psi ,S_1,S_2,I,T,R)d\psi \end{aligned}$$

By, Lagrangian polynomial piece-wise interpolation, we get

97 $$\begin{aligned} \begin{array}{c} S_1^{b+1}=S_1^{0}+\frac{\phi t^{\phi -1}_{b}(1-\gamma )}{AB(\gamma )}g_{1}(t_{b},S_1^{b},S_2^{b},I^{b},T^{b},R^{b})+\frac{\gamma (\Delta t)^{\gamma }}{AB(\gamma )\Gamma (\gamma +2)}\\ \sum \limits ^{b}_{c=0}\left[t^{\phi -1}_{c}g_{1}(t_{c},S_1^{c},S_2^{c},I^{c},T^{c},R^{c})\times ((b+1-c)^{\gamma }(b-c+\gamma +2)-(b-c)^{\gamma }(2+2\gamma +b-c))\right.\\ \left.-t^{\phi -1}_{c-1}g_{1}(t_{c-1},S_1^{c-1},S_2^{c-1},I^{c-1},T^{c-1},R^{c-1})\times ((1+b-c)^{\gamma +1}-(b-c)^{\gamma }(1+\gamma +b-c))\right] \end{array} \end{aligned}$$

98 $$\begin{aligned} \begin{array}{c} S_2^{b+1}=S_2^{0}+\frac{\phi t^{\phi -1}_{b}(1-\gamma )}{AB(\gamma )}g_{2}(t_{b},S_1^{b},S_2^{b},I^{b},T^{b},R^{b})+\frac{\gamma (\Delta t)^{\gamma }}{AB(\gamma )\Gamma (\gamma +2)}\\ \sum \limits ^{b}_{c=0}\left[t^{\phi -1}_{c}g_{2}(t_{c},S_1^{c},S_2^{c},I^{c},T^{c},R^{c})\times ((b+1-c)^{\gamma }(b-c+\gamma +2)-(b-c)^{\gamma }(2+2\gamma +b-c))\right.\\ \left.-t^{\phi -1}_{c-1}g_{1}(t_{c-1},S_1^{c-1},S_2^{c-1},I^{c-1},T^{c-1},R^{c-1})\times ((1+b-c)^{\gamma +1}-(b-c)^{\gamma }(1+\gamma +b-c))\right] \end{array} \end{aligned}$$

99 $$\begin{aligned} \begin{array}{c} I^{b+1}=I^{0}+\frac{\phi t^{\phi -1}_{b}(1-\gamma )}{AB(\gamma )}g_{3}(t_{b},S_1^{b},S_2^{b},I^{b},T^{b},R^{b})+\frac{\gamma (\Delta t)^{\gamma }}{AB(\gamma )\Gamma (\gamma +2)}\\ \sum \limits ^{b}_{c=0}\left[t^{\phi -1}_{c}g_{3}(t_{c},S_1^{c},S_2^{c},I^{c},T^{c},R^{c})\times ((b+1-c)^{\gamma }(b-c+\gamma +2)-(b-c)^{\gamma }(2+2\gamma +b-c))\right.\\ \left.-t^{\phi -1}_{c-1}g_{1}(t_{c-1},S_1^{c-1},S_2^{c-1},I^{c-1},T^{c-1},R^{c-1})\times ((1+b-c)^{\gamma +1}-(b-c)^{\gamma }(1+\gamma +b-c))\right] \end{array} \end{aligned}$$

100 $$\begin{aligned} \begin{array}{c} T^{b+1}=T^{0}+\frac{\phi t^{\phi -1}_{b}(1-\gamma )}{AB(\gamma )}g_{4}(t_{b},S_1^{b},S_2^{b},I^{b},T^{b},R^{b})+\frac{\gamma (\Delta t)^{\gamma }}{AB(\gamma )\Gamma (\gamma +2)}\\ \sum \limits ^{b}_{c=0}\left[t^{\phi -1}_{c}g_{1}(t_{c},S_1^{c},S_2^{c},I^{c},T^{c},R^{c})\times ((b+1-c)^{\gamma }(b-c+\gamma +2)-(b-c)^{\gamma }(2+2\gamma +b-c))\right.\\ \left.-t^{\phi -1}_{c-1}g_{4}(t_{c-1},S_1^{c-1},S_2^{c-1},I^{c-1},T^{c-1},R^{c-1})\times ((1+b-c)^{\gamma +1}-(b-c)^{\gamma }(1+\gamma +b-c))\right] \end{array} \end{aligned}$$

101 $$\begin{aligned} \begin{array}{c} R^{b+1}=R^{0}+\frac{\phi t^{\phi -1}_{b}(1-\gamma )}{AB(\gamma )}g_{5}(t_{b},S_1^{b},S_2^{b},I^{b},T^{b},R^{b})+\frac{\gamma (\Delta t)^{\gamma }}{AB(\gamma )\Gamma (\gamma +2)}\\ \sum \limits ^{b}_{c=0}\left[t^{\phi -1}_{c}g_{1}(t_{c},S_1^{c},S_2^{c},I^{c},T^{c},R^{c})\times ((b+1-c)^{\gamma }(b-c+\gamma +2)-(b-c)^{\gamma }(2+2\gamma +b-c))\right.\\ \left.-t^{\phi -1}_{c-1}g_{5}(t_{c-1},S_1^{c-1},S_2^{c-1},I^{c-1},T^{c-1},R^{c-1})\times ((1+b-c)^{\gamma +1}-(b-c)^{\gamma }(1+\gamma +b-c))\right] \end{array} \end{aligned}$$

## Numerical results and discussions

**Fig. 1:**
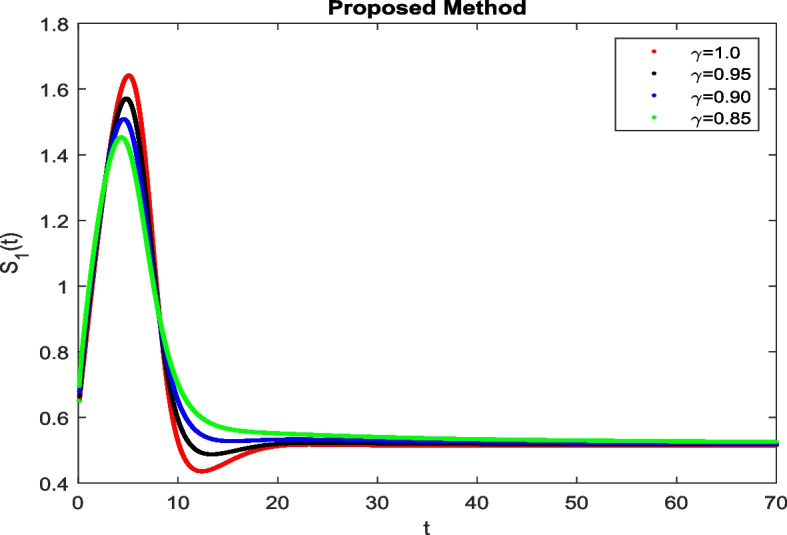
Solution of $S_{1}(t)$ for different fractional values

**Fig. 2:**
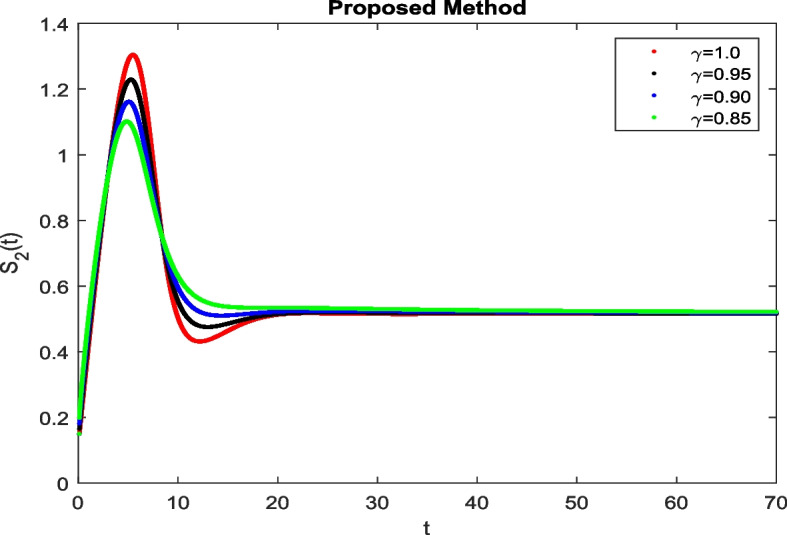
Solution of $S_{2}(t)$ for different fractional values

**Fig. 3:**
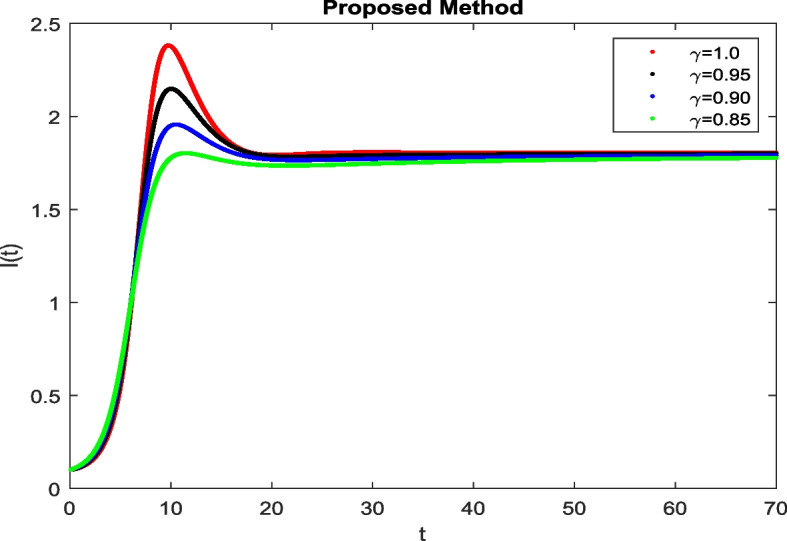
Solution of *I*(*t*) for different fractional values

**Fig. 4:**
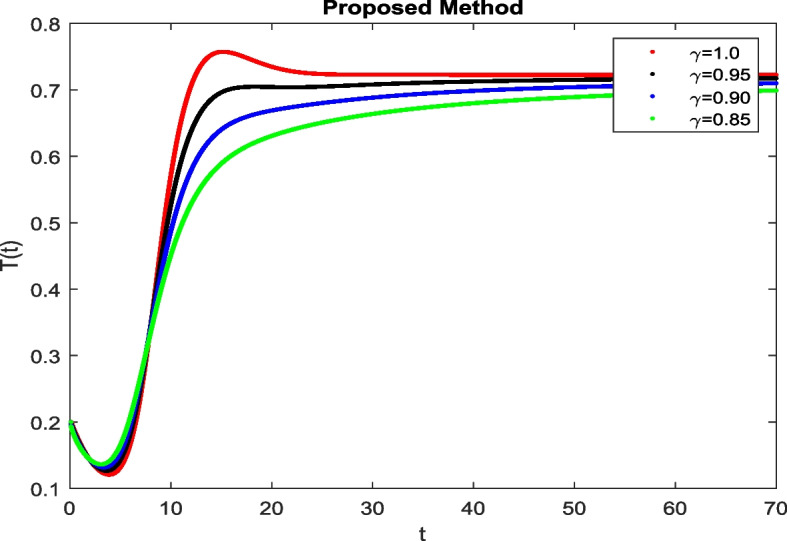
Solution of *T*(*t*) for different fractional values

**Fig. 5:**
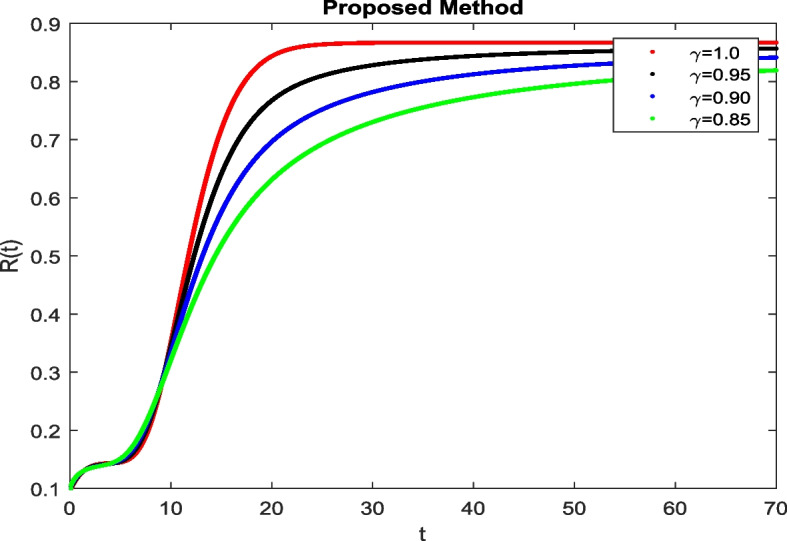
Solution of *R*(*t*) for different fractional values

**Fig. 6:**
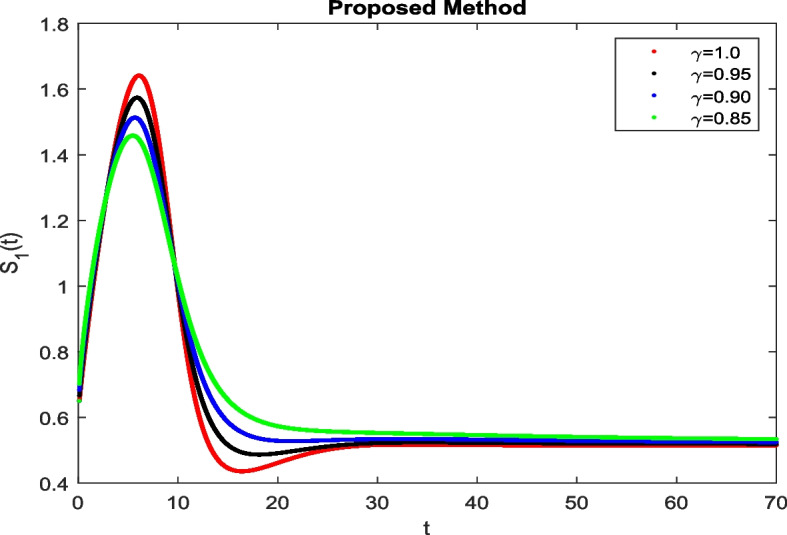
Solution of $S_{1}(t)$ for Fractal Fractional Operator with dimension 0.9

**Fig. 7:**
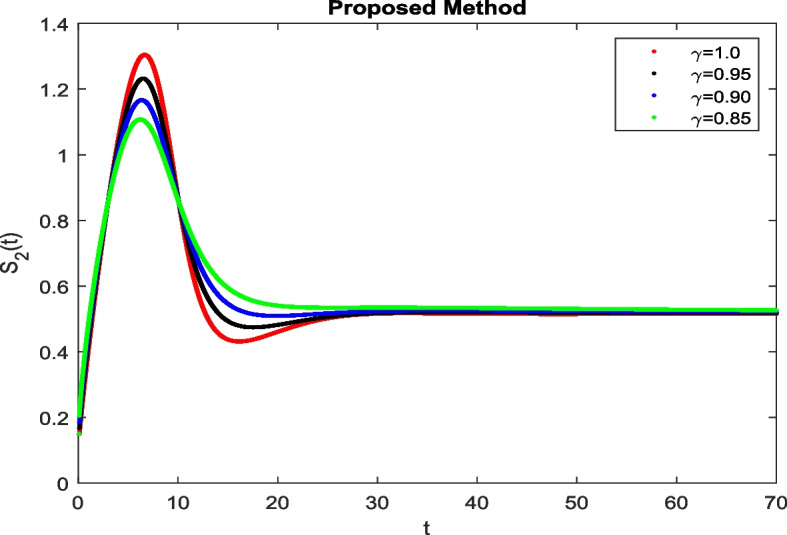
Solution of $S_{2}(t)$ for Fractal Fractional Operator with dimension 0.9

**Fig. 8:**
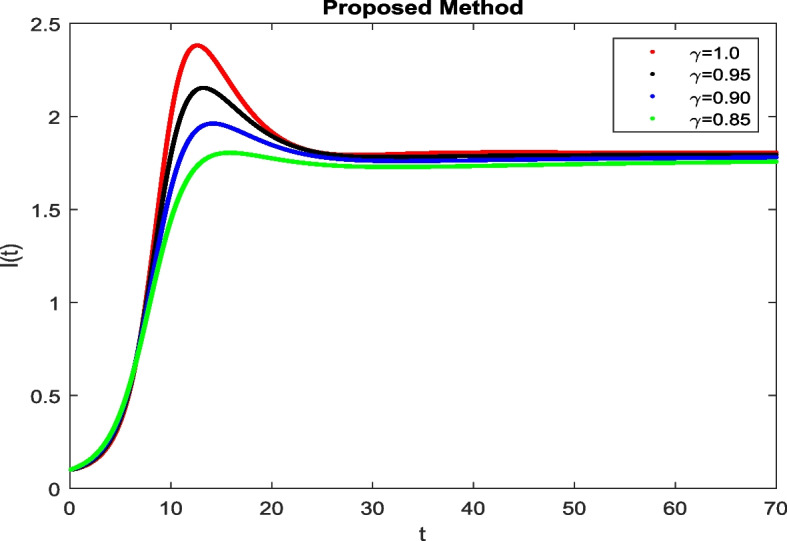
Solution of *I*(*t*) for Fractal Fractional Operator with dimension 0.9

**Fig. 9:**
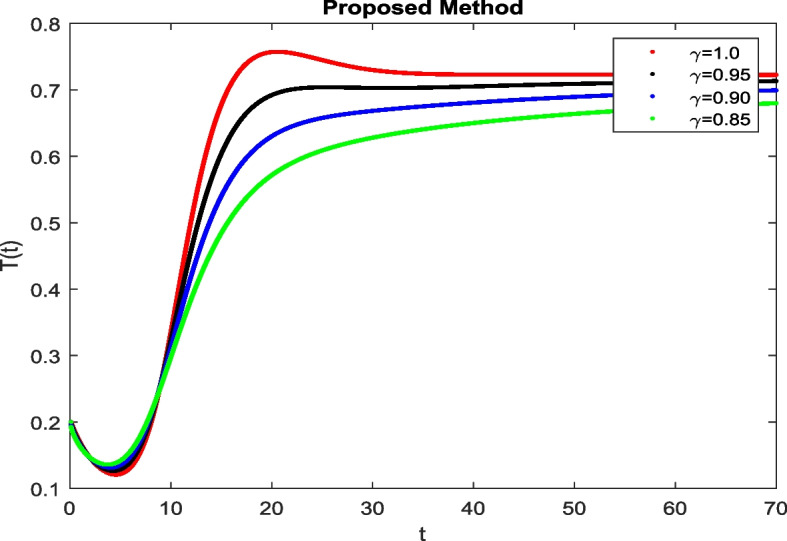
Solution of *T*(*t*) for Fractal Fractional Operator with dimension 0.9

**Fig. 10:**
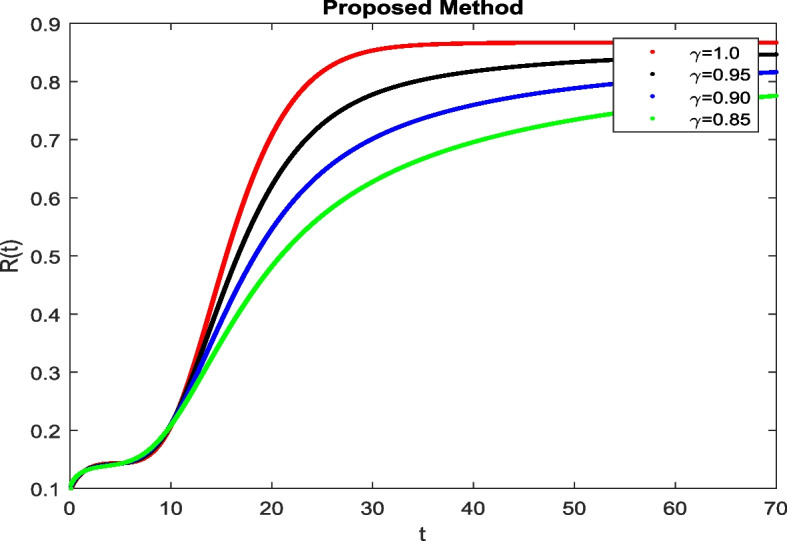
Solution of *R*(*t*) for Fractal Fractional Operator with dimension 0.9

In this section, To identify the potential transmission of COVID-19 with different age groups in the Community, the proposed fractional-order model is presented to analyze with simulations. The effectiveness of the obtained theoretical outcomes are established by using advanced techniques. Intrusting findings are achieved by implementing the non-integer parametric choices of the COVID-19 system. MATLAB coding is employed to find the numerical simulation for fractional order COVID-19 model using different fractional values. Uninfected and old age(which have some sickness) population increases strictly but after certain time it reduces in the same way then come at stable position as can be seen in Figs. 1 and 2 respectively. Infected individual rises but after certain time it approach to stable position due to increase in treatment which also provide increase in recovered individual as can be observed from Figs. 3, 4 and 5 respectively by using Atangana Toufik technique. Similar behavior can be seen in Figs. 6, 7, 8, 9 and 10 by using fractal fractional technique with dimension 0.9. But we can observe from figures easily that it provide us better and efficient results, and effect of fractional parameter as well more clear when using fractal fractional technique with dimension 0.9. In Figs. 1, 2, 3, 4, 5, 6, 7, 8, 9 and 10, solution for all compartments comes according to desired value by decreasing the fractional values using both techniques Atangana Toufik and fractal fractional with minor effects. It can be easily deduce that, we can get more better results by using fractal fractional technique by reducing fractional values as well as reducing dimensions, because we approach stable position faster and deduce that recovered start rising and infected become stable after certain time due to treatment for both age groups. Also, we find that the Fractal Fractional technique provide reliable findings for all compartment according to steady state at non-integer fractional values as compare to integer values by reducing its dimensions. It is also observed that researchers may predicts what should happen in future by this research.

## Conclusion

In this article, we consider Hybrid NAR-RBFs Networks for COVID-19 model with fractional operator like Atangana-Toufik and Fractal Fractional Operator which is analyzed to see its effects. We have presented some advises to control this virus so that our community can overcome this pandemic. The dangerous corona virus and the deadly epidemic of Hybrid NAR-RBFs Networks for COVID-19 disease in today’s pandemic have caused millions of deaths to date. Further, boundedness and stability are verified as well as unique solution of the proposed system is verified to check the efficiency of the system and steady state solutions. The obtained solutions demonstrate a reliable findings to control the terrible effect of COVID-19 with help of advanced techniques for the different age groups and to eliminate the death killer factor in the community. The predictions are made that, our solution created from advanced techniques Atangana-Toufik and fractal fractional which are effective by reducing the fractional values because all graphical representations behavior approaches to steady state. These representations of all compartments demonstrate that how COVID-19 effects behaves by change the parameter values, also provide continuous monitoring of spread of disease in different age groups. Simulation makes it more simple and easy to see how individuals with different age groups effected by COVID-19 with the passage of time. Comparison is done to see the efficiency of results and change in effects. The authors believe that the synchronization developed systems and displayed figures of the COVID-19 under Hybrid NAR-RBFs Networks system have revealed complex dynamical behaviors that are not achieved earlier. Such kind of research provide more significant tactics to control or overcome COVID-19 effects as well as help in decision making. In future the simulations with different arrangements of fractional values can be implemented to gain a sampling of conceivable behaviors in the framework of dynamical systems. Furthermore, the matter of local and global stability for such model with COVID-19 system, which is an significant issue in our next research work.

## Data Availability

Data will be provided on request to the corresponding author.
